# Dog ownership, physical activity, loneliness and mental health: a comparison of older adult and younger adult companion animal owners

**DOI:** 10.1186/s40359-024-02104-x

**Published:** 2024-11-01

**Authors:** Kirrily Zablan, Glenn Melvin, Alexa Hayley

**Affiliations:** School of Psychology, Faculty of Health, Deakin University, Waurn Ponds Campus, 75 Pigdons Road, Waurn Ponds, VIC 3216 Australia

**Keywords:** Companion animals, Pet ownership, Physical activity, Dog walking, Mental health, Loneliness, Older adults

## Abstract

**Background and aim:**

Dog ownership has been suggested as an intervention to increase physical activity and improve mental health, but few studies have investigated the relationships between dog ownership, physical activity, and mental health outcomes together. This study aimed to (1) investigate whether dog ownership, CA-related physical activity, and non-CA-related physical activity were explanatory variables for the relationships between CA ownership, depression, and anxiety via loneliness and (2) examine whether the relationships between these variables differed for older adult CA owners compared to younger adult CA owners.

**Method:**

Participants were Australian CA owners from the community (*N* = 588, 76.3% female) aged 18–84 years (*M* = 55.34, *SD* = 14.90). A cross-sectional design and online/phone survey methodology were used.

**Results:**

Path analysis showed that dog owners (compared to owners of other CA types) engaged in higher levels of both CA-related and non-CA-related physical activity, but only non-CA-related physical activity was associated with mental health outcomes. Multigroup moderation analysis showed that older adult and younger adult CA owners experienced similar moderate levels of loneliness, while in younger adults this moderate loneliness was associated with higher levels of depression and anxiety as compared to older adults.

**Conclusion:**

Our findings indicate people who choose to own dogs over other CA types engage in more active lifestyles, but it is the physical activity they perform independently of their dog that is associated with less loneliness and greater mental health. Members of the public should not be universally encouraged by health or other professionals to own a dog to support their mental health based on a belief that dog ownership leads to beneficial physical activity.

**Supplementary Information:**

The online version contains supplementary material available at 10.1186/s40359-024-02104-x.

The notion that companion animal ownership (CAO) can reduce loneliness and improve mental health outcomes is not a new one. Companion animal (CA) owners have long perceived their CAs make them happier and healthier [2]. This belief was reinforced during the COVID-19 pandemic as CA researchers and medical professionals released opinion pieces publicising the ‘benefits’ of CAO for mental health [25, 30], and people bought and adopted CAs at increased rates to cope with pandemic lockdowns [41, 51]. Qualitative research conducted during the pandemic reflects this positive public sentiment [51, 54, 64], but quantitative findings have largely disputed the ‘feel-good’ story of CAO and pandemic wellbeing.

Quantitative CA COVID-19 studies comparing the loneliness, mental health, and wellbeing of CA owners and non-CA owners reported a non-significant effect of CAO [e.g. 13, 20, 28], or negative effect of CAO [e.g. 3; 34, 42], on these outcomes. Although only a small number of studies reported CAO being associated with less loneliness, better mental health, or improved wellbeing [e.g. 6, 48, 58], these positive findings were over-represented in media articles during the pandemic, providing a biased view of the CAO COVID-19 literature [10, 43]. Taken as a whole, the body of literature instead indicates CAO did not significantly reduce loneliness and improve mental health during the COVID-19 pandemic.

## Dog ownership, physical activity, loneliness, and mental health

Human-animal interactions (HAI) researchers have proposed explanations for the ‘pet effect paradox’ [31], where qualitative evidence provides compelling support for CAO when sampling CA owners, but quantitative evidence indicates no significant relationship between CAO and mental health. One possible explanation is that merely comparing CA owners and non-owners does not account for CA-related variables possibly influencing the relationship between CAO and mental health, such as the type of CA owned, or whether a CA promotes increased physical activity [32]. The CAO COVID-19 evidence suggests CA type and physical activity may be meaningful explanatory variables when considering the relationship between CAO and mental health [58, 58]. Two of the studies reporting a positive association between CAO and mental health outcomes included physical activity as a variable of interest [6, 58]. Bohn and colleagues [6] found dog ownership and physical activity to be independently associated with lower depression during lockdown, while cat and bird ownership was not significantly associated with depression, demonstrating mixed results across CA types. Tan and colleagues (2021) investigated CAO and physical activity during lockdown in Singapore, finding CA owners reported higher mild-intensity physical activity and emotional wellbeing than non-owners. These findings suggest that CA type and physical activity may be unexpectantly impacting CAO comparison studies.

Reviewing the proposed theories of HAI also provides guidance as to which individual differences could explain why the effect of CAO on loneliness and mental health outcomes varies. These theories of HAI attempt to explain *how* CAO may improve human mental health, including the companionship [44], stress reduction [23], attachment and oxytocin [5], social catalyst [61], exposure to green spaces [65], and physical activity theories [19].

Dog ownership has been a focus of HAI research due to the unique care dogs require. The exercise requirements of dogs far exceed those of other common CA types typically kept in the home, including cats, fish, birds, small mammals, and reptiles [15]. Dogs usually require exercise outside the home, not only for their physical health, but also for mental stimulation [15]. A wealth of evidence suggests dog ownership results in increased recreational walking and increased overall physical activity for dog owners [11, 60], and there is clear evidence indicating physical activity strongly predicts both physical and mental health [1].

Loneliness is a major contributor to poor mental health and longitudinal research has demonstrated loneliness as predicting the later development of depression [29]. Although loneliness can be conceptualised as an indicator of unmet social needs and a motivator to implement steps to increase social connection [45], during the COVID-19 pandemic, the ability to put these steps into action were significantly restricted by public health directives [4], resulting in increased depression and anxiety for global citizens during the pandemic (Robinson et al., 2022). Dog walking may benefit mental health and reduce loneliness via a social catalyst effect, as walking a dog increases the frequency of chance social interactions with strangers [40, 61]. During COVID-19 such chance ‘COVID-safe’ social encounters were at times the only social contact dog owners experienced [64], and studies showed increased dog walking during COVID-19 resulted in reduced loneliness [9, 35].

Dog walking may also expose owners to natural outdoor environments, or green spaces. Exposure to green spaces has been cited as a positive contributor to mental health, in addition to the benefits of physical activity [59]. The physical activity, social catalyst, and exposure to green spaces theories all provide indications dog ownership may predict reduced loneliness and improved mental health via dog walking.

Although the theoretical basis for relationships between dog ownership, physical activity, and mental health is strong, the research evidence is not so clear. This is, in part, because few studies investigate the two relationships of interest; whether dog ownership predicts increased physical activity, and whether this increased physical activity predicts mental health outcomes. Those that do, have reported mixed findings. Dunn and colleagues [21] found dog ownership was associated with increased physical activity, but not depression or loneliness among cardiac patients. Conversely, studies conducted in Singapore suggested CA owners/caregivers engaged in more physical activity, and had higher scores on measures of emotional wellbeing, social functioning, and energy [26, 58]. Other studies investigated whether dog walking predicted mental health outcomes, but not whether dog ownership engaged in more physical activity or walking than non-dog ownership. Two of these studies found dog walking was associated with reduced loneliness [9, 35], while one found no significant association between dog walking and loneliness [47]. When considering depression outcomes, evidence was similarly mixed, with one study reporting no significant relationship between dog walking and depression [18], while one study found dog walking was associated with reduced depression [38]. It is surprising, given the large research area of dog ownership and physical activity, that so few studies investigate dog ownership, physical activity, loneliness, and mental health outcomes together.

### Older adult dog ownership, physical activity, loneliness and mental health

Older adult loneliness and mental health have been priorities of mental health research during COVID-19 [36, 62]. Research evidence suggests older adults did experience increased rates of loneliness [57], depression [7], and anxiety [27] during the pandemic, although people within other demographic groups, such as younger adults and women, appeared to experience similar or worse mental health outcomes [8, 37]. Older adults have also been a particular focus of research investigating dog ownership and physical activity. Multiple studies have demonstrated older adult dog owners as engaging in significantly more walking than non-dog owners [19, 22]. It is possible the physical activity promoted by dog ownership is more beneficial for older adults, due to older adults engaging in less overall physical activity than younger adults [12].

Our study will add to the literature by being the first to investigate dog ownership, CA-related physical activity such as dog walking, physical activity not conducted with a CA, and mental health outcomes together. This will provide insights into the types of physical activity associated with mental health outcomes, and whether physical activity conducted with or without a CA is more beneficial. Our study is also the first to investigate whether the relationships between dog ownership, physical activity, and mental ill health are stronger for older adults compared to younger adults.

### Aims and hypotheses

This study aimed to (1) investigate whether dog ownership, CA-related physical activity, and non-CA-related physical activity were meaningful explanatory variables for the relationships between CAO, depression, and anxiety via loneliness and (2) examine whether the relationships between these variables differed for older adult CA owners compared to younger adult CA owners.

**Fig. 1:**
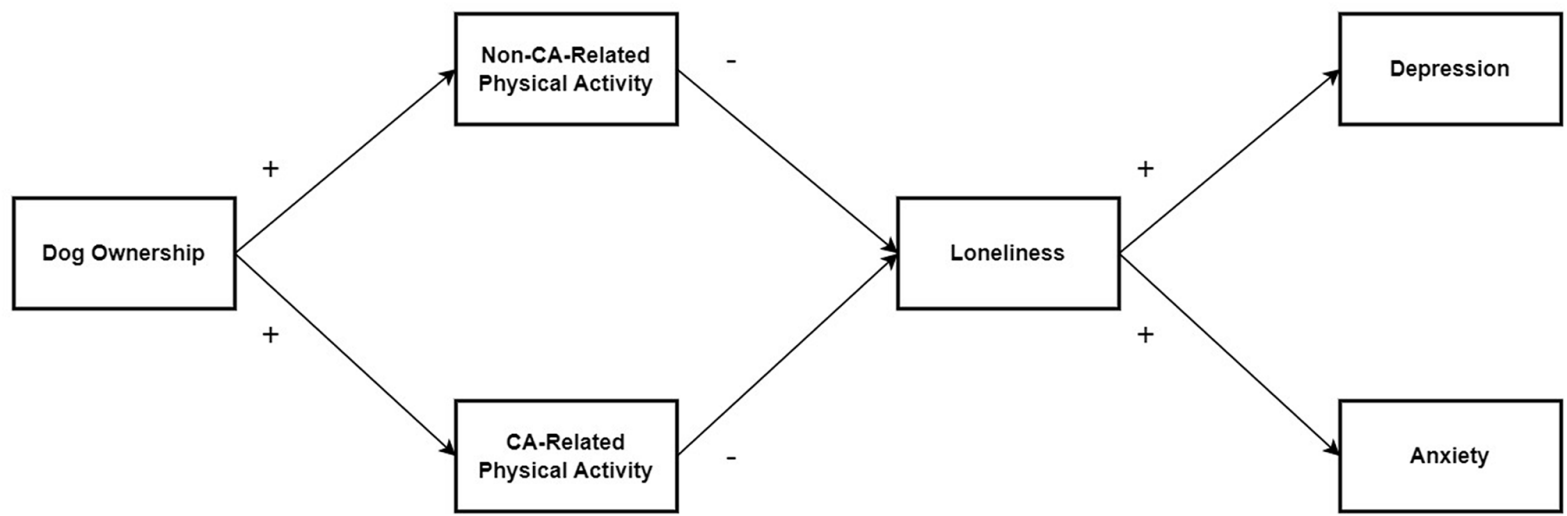
Conceptual model of associations between dog ownership, non-CA-related physical activity, CA-related physical activity, loneliness, depression, and anxiety *Note*. + = hypothesised positive association, - = hypothesised negative association. CA = Companion animal

The hypothesised relationships are displayed in Fig. 1. We hypothesised these relationships would be stronger for older adult CA owners compared to younger adult CA owners.

## Method

### Human ethics and consent to participate

This study was approved by the Deakin University Human Research Ethics Committee (2020–231) and conducted in accordance with the Australian Code for the Responsible Conduct of Research, 2018. Informed consent was obtained from all participants.

### Participants

Eligibility criteria required participants be: (1) an Australian citizen or permanent resident; (2) aged 18 years or older; and (3) currently own at least one CA. Participants (*N* = 588, 76.3% female) were a convenience sample of CA owners from the Australian community, aged 18 to 84 years (*M* = 55.34, *SD* = 14.90). The overall sample consisted of two subsamples, older adult CA owners aged 65 years and over (*n* = 186, *M* = 70.7 years, *SD* = 4.4 years, 88.4% female) and younger adult CA owners aged 64 and under (*n* = 402, *M* = 48.4 years, *SD* = 12.7 years, 71.4% female). Participant demographics for both the older adult and younger adult subsamples, including COVID-19 lifestyle impacts, are presented in Table 1.

**Table 1:** Participant characteristics for older adult (65 + years; *n* = 186) and younger adult (> 65 years; *n* = 402) companion animal owners

Characteristic	65 + Sample	U65 Sample
	*n* (%)	*n* (%)
Age		
* M* (*SD*)	70.7 (4.4)	48.4 (12.7)
Sex		
Female	160 (88.4)	287 (71.4)
Male	20 (11.0)	113 (28.1)
Another sex	1 (0.6)	2 (0.5)
Education Level		
Some University Education	81 (43.5)	195 (48.5)
No University Education	105 (56.5)	207 (51.5)
CA Type Owned		
Dog	129 (71.3)	279 (69.4)
Cat	77 (42.5)	171 (42.5)
Small mammal	1 (0.54)	18 (4.5)
Fish	12 (6.6)	42 (10.4)
Bird	28 (15.1)	58 (14.4)
Horse/Pony	0 (0.0)	12 (3.0)
Livestock as CA	14 (7.5)	13 (3.2)
Wildlife as CA	6 (3.2)	1 (0.2)
Relationship Status		
Not currently in a relationship	88 (47.3)	154 (38.3)
In a relationship	91 (48.9)	243 (60.4)
Not stated	7 (3.8)	5 (1.2)
Living Situation		
Lives alone	89 (47.8)	95 (23.6)
Lives with others	92 (49.5)	307 (76.4)
Not stated	5 (2.7)	0 (0.0)
Employment Status		
Employed	24 (12.9)	271 (67.4)
Not employed	11 (5.9)	89 (22.1)
Retired	151 (81.2)	42 (10.4)
COVID-19 Impacts		
Made redundant/contract not renewed	4 (2.2)	21 (5.2)
Reduced work hours	4 (2.2)	45 (11.2)
Some or all work conducted from home	12 (6.5)	122 (30.3)
Quarantined within previous 4 months	24 (13.3)	70 (17.4)

*Note* CA = Companion animal, *M* = mean, *SD* = standard deviation

### Design

A cross-sectional online survey methodology was used. Data was collected over a 3-month period during the COVID-19 pandemic from November 1 2020 to January 31 2021. The data was timepoint 1 of a larger 12-month longitudinal survey dataset.

### Measures

#### Demographics

Participant age, sex (i.e. male, female, non-binary/another sex), state/territory, highest level of education, employment status, relationship status, living situation, CA type/s owned, and COVID-19 lifestyle impacts including redundancy, reduced work hours, work from home, and quarantine.

#### Dog ownership

Dog ownership was a dichotomous measure where those who did not own a dog but owned another CA type were coded as 0, while those who owned a dog were coded as 1. Those owning a dog and another CA type were coded as 1 (dog owners).

#### Depression, anxiety, and stress scale – 21 (DASS-21) [39]

The DASS-21 consists of three self-report subscales measuring symptoms of depression, anxiety, and stress during the past four weeks. Participants responded to each item using a 4-point Likert-type scale (0 = ‘never’, 1 = ‘sometimes’, 2 = ‘often’, 3 = ‘almost always’), with a possible range of scores per subscale being 0 to 21. Only the depression and anxiety subscales were used in the current study. Internal consistency of the depression subscale was α = 0.93 (younger adult subsample), α = 0.94 (older adult subsample) and the anxiety subscale was α = 0.66 (younger adult subsample), α = 0.86 (older adult subsample).

#### UCLA loneliness scale – version 3 (UCLA) [53]

The UCLA is a 20-item scale designed to measure one’s subjective feelings of loneliness and social isolation. Participants responded to each item on a 4-point Likert scale (1 = ‘never’, 2 = ‘rarely’, 3 = ‘sometimes’, 4 = ‘often’) with a minimum score of 20 and a maximum score of 80. Internal consistency was observed as α = 0.95 in the current study.

#### Physical activity (DASS-21) [39]

CA-related physical activity and non-CA-related physical activity were measured using the following original items developed for this study: ‘*Approximately how many hours of pet-related physical activity do you participate in per week? (e.g.*, *walking a dog*, *cleaning up after your pet etc.)’* and *‘Approximately how many hours of physical activity do you participate in per week? (e.g.*, *walking*, *gardening*, *running*, *strength exercises*, *sports etc.)’.* Participants responded to each item on a 4-point Likert scale (1 = ‘less than one hour’, 2 = ‘1 to 3 hours’, 3 = ‘3 to 6 hours’, 4 = ‘more than 6 hours’).

### Procedure

Participants were recruited for a mixed-methods longitudinal survey using two recruitment strategies to allow older adults with varied digital literacy to participate; (1) paid and unpaid social media advertisements (Facebook, Instagram), with the advertisement’s URL directed to the online survey hosted on survey platform Qualtrics; and (2) 450 paper flyers distributed at 12 physical sites visited by older adults (i.e. retirement villages, Returned Service League, and Rotary Club) inviting eligible participants to complete the online or phone survey. On completion of the survey (approximately 30 min), respondents were invited to enter a prize draw to win one of two $50 e-gift vouchers. Data analysis was completed using IBM SPSS Statistics 28 and IBM SPSS AMOS 26 software.

### Data analysis plan

Preliminary analyses included descriptive statistics for age, sex, education level, employment status, state/territory, relationship status, living situation, and type of CA owned, as well as for COVID-19 lifestyle impacts including lockdown, redundancy, reduced work hours, working from home, and quarantine. Bivariate correlation analyses were conducted to identify linear associations among the variables prior to path analysis. Independent sample *t*-tests were performed to determine age-based differences for loneliness, depression, and anxiety. Path analysis, employing maximum likelihood estimation, was used to identify the relationships between variables of interest. One conceptual path analysis was used to assess the relationships between dog ownership, CA-related physical activity, non-CA-related physical activity, loneliness, depression, and anxiety. This analysis was conducted twice, one on a subsample of older adult CA owners and one on a subsample of younger adult CA owners. Path analysis global fit was calculated using the criteria: χ²(*df*), *p* > .05; χ²/*df* < 3; root mean square error of approximation (RMSEA < 0.08, <0.11 for samples < 200); comparative fit index (CFI > 0.95) and incremental fit index (IFI > 0.95) [33]. A *p*-value < 0.05 was considered significant for path analysis local fit. AMOS was used to assess relationships between variables using a path model drawn from theory (see Fig. 1). The model conducted on each subsample was optimised by removing non-significant paths (*p* > .05) and addition of paths indicated by modification indices (MI > 3.84) [33]. Multigroup analysis was conducted to compare the standardised coefficients for each path of the conceptual model between each subsample to determine whether significant differences existed between the older adult CA owner and younger adult CA owner path analyses. The minimum necessary sample size for statistical significance at the *p* < .05 level, using the 10:1 rule for path analysis is 120 per path analysis [33]. Sample sizes for each subsample met these criteria.

## Results

### Preliminary analyses

Of the initial 959 survey responses from CA owners, *n* = 371 cases were removed due to incomplete responses, leaving *N* = 588 participants. Descriptive statistics for outcome measures depression and anxiety are summarised in Table 2. Descriptive statistics and *t*-tests results are also presented in Table 2. As shown, older adults scored significantly lower on depression and anxiety than younger adults (*p*’s < 0.001). There was no significant difference between older adults and younger adults on loneliness (*p* = .50). Table 3 shows the results of bivariate correlation analyses between the study variables for both older adult CA owners and younger adult CA owners. Bivariate correlations indicated several significant, small to medium, associations between variables of interest from *r* = − .12 to *r* = .37 [14]. Absence of multicollinearity was confirmed with all associations among outcome variables *r* < .80. Bivariate correlations indicated for older adult CA owners, dog ownership (versus ownership of another CA type) was significantly negatively associated with anxiety (*r* = − .16), but not associated with loneliness and depression. For younger adult CA owners, dog ownership (versus ownership of another CA type) was significantly negatively associated with loneliness (*r* = − .13), and depression (*r* = − .12), but not associated with anxiety. For both older adults and younger adults, dog ownership (versus ownership of another CA type) was positively associated with CA-related physical activity and non-CA-related physical activity, with small to medium effect sizes (*r* = .19 to *r* = .37) [14]. CA-related physical activity was significantly negatively associated with depression (*r* = − .19) and anxiety (*r* = − .20) in older adult CA owners, but not in younger adult CA owners. In older adult CA owners, non-CA-related physical activity was significantly negatively associated with depression (*r* = − .19) and anxiety (*r* = − .20), while in younger adult CA owners, non-CA-related physical activity was significantly negatively associated with loneliness (*r* = − .12) and depression (*r* = − .12).

### Hypothesis testing

#### Loneliness depression and anxiety in older adult CA owners

**Fig. 2:**
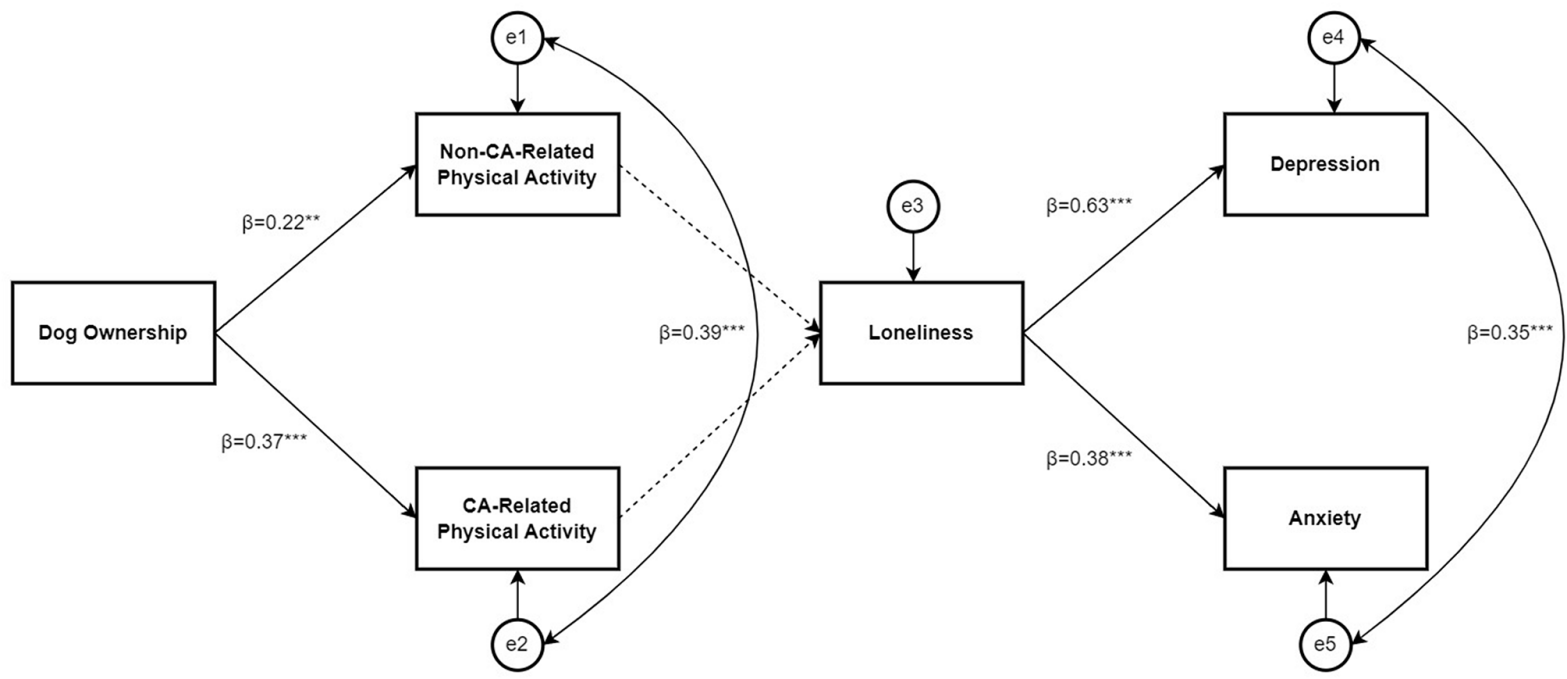
Testing the relationships between dog ownership, non-CA-related physical activity, CA-related physical activity, loneliness, depression, and anxiety, on a sample of older adult CA owners aged 65 years and over. *Note*. ***p<.001, **p<.01, * *p*<.05. →= Non-significant path. Coefficients are standardised regression weights. CA = Companion animal. RMSEA=0.06; CFI=0.97; IFI=0.97; χ²=15.54, df=9, *p*=.08, χ²/df=1.73. e1 – e5 = residual error terms reflecting the unexplained variance and measurement error for each variable

Figure 1 shows the conceptual model while Fig. 2 shows the final model with good model fit (RMSEA = 0.06; CFI = 0.97; IFI = 0.97; χ² = 15.54, *df* = 9, *p* = .08, χ²/*df* = 1.73). Table 4 shows the fit statistics for alternative models. The final model (Fig. 2) demonstrates dog ownership was significantly positively associated with non-CA-related physical activity (β = 0.22, *p* = .003) and CA-related physical activity (β = 0.37, *p* < .001), while these were not significantly associated with loneliness resulting in no complete pathways in the path analysis. Dog ownership explained 5% of the variance in non-CA-related physical activity (R² = 0.05), and 14% of the variance in CA-related physical activity (R² = 0.14). The error terms for non-CA-related physical activity and CA-related physical activity were significantly positively associated (β = 0.39, *p* < .001), indicating possible overlap in these constructs. Loneliness was significantly positively associated with depression (β = 0.63, *p* < .001), and anxiety (β = 0.38, *p* < .001), with loneliness explaining 40% of the variance in depression (R² = 0.40), and 14% of the variance in anxiety (R² = 0.14).

**Table 2:** Descriptive statistics, symptom severity on the DASS-21 depression and anxiety subscales for older adult (65+) companion animal owners (*n* = 186) and younger adult (U65) companion animal owners (*n* = 402), and results of T-tests examining age differences on loneliness, depression, and anxiety levels for companion animal owners

Outcome Measures	65+				Outcome Measures	U65				t	df	*p*
	M (SD)	α	Symptom Severity	*n* (%)		M (SD)	α	Symptom Severity	*n* (%)			
Depression	4.06 (4.52)	0.93	Normal	112 (61.9)	Depression	6.37 (5.84)	0.94	Normal	199 (49.5)	4.79	601	< 0.001
			Mild	28 (15.5)				Mild	38 (9.5)			
			Moderate	22 (12.2)				Moderate	71 (17.7)			
			Severe	10 (5.5)				Severe	33 (8.2)			
			Extremely Severe	9 (5.0)				Extremely Severe	61 (15.2)			
Anxiety	1.63 (2.09)	0.66	Normal	154 (85.1)	Anxiety	3.32 (3.89)	0.86	Normal	264 (65.7)	5.57	601	< 0.001
			Mild	12 (6.6)				Mild	47 (11.7)			
			Moderate	11 (6.1)				Moderate	33 (8.2)			
			Severe	4 (2.2)				Severe	28 (7.0)			
			Extremely Severe	0 (0.0)				Extremely Severe	30 (7.5)			
Loneliness	2.10 (0.65)	0.95			Loneliness	2.40 (0.67)	0.95			5.1	601	0.5
CA Related Physical Activity	2.94 (1.10)				CA Related Physical Activity	2.70 (1.09)						
Non-CA Related Physical Activity	3.00 (1.02)				Non-CA Related Physical Activity	2.75 (1.06)						

*Note* M = mean, *SD* = standard deviation, α = Cronbach’s alpha, CA = Companion animal. ***p < 001, **p < .01, *p < .05

**Table 3:** Bivariate correlations for older adult (65+) companion animal owners and younger adult (U65) companion animal owners

	Variable	Older Adult CA Owners					Younger Adult CA Owners				
		1	2	3	4	5	1	2	3	4	5
1.	Dog Ownership	-					-				
2.	CA-Related Physical Activity	0.37**	-				0.33**	-			
3.	Non-CA Related Physical Activity	0.22**	0.44**	-			0.19**	0.57**	-		
4.	Loneliness	0.01	0.02	− 0.10	-		− 0.13*	− 0.09	− 0.12*	-	
5.	Depression	− 0.04	− 0.19*	− 0.19*	0.63**	-	− 0.12*	− 0.06	− 0.12*	0.73**	-
6.	Anxiety	− 0.16*	− 0.20**	− 0.20**	0.38**	0.49**	− 0.08	− 0.03	− 0.06	0.51**	0.66**

*Note* **p < .01, *p < .05. CA = Companion animal

**Table 4:** Fit statistics for alternative models

Model	χ²	df	*p*	χ²/df	RMSEA	CFI	IFI
1	67.16	9	0.000	7.46	0.19	0.72	0.73
2	68.10	10	0.000	6.81	0.18	0.72	0.73
3	69.99	11	0.000	6.36	0.17	0.72	0.72
4	39.66	10	0.000	3.97	0.13	0.86	0.86
5	15.54	9	0.08	1.73	0.06	0.97	0.97

*Note* RMSEA = root mean square error of estimation; CFI = comparative fit index; IFI = incremental fit index

#### Loneliness Depression and Anxiety in Younger Adult CA Owners

**Fig. 3:**
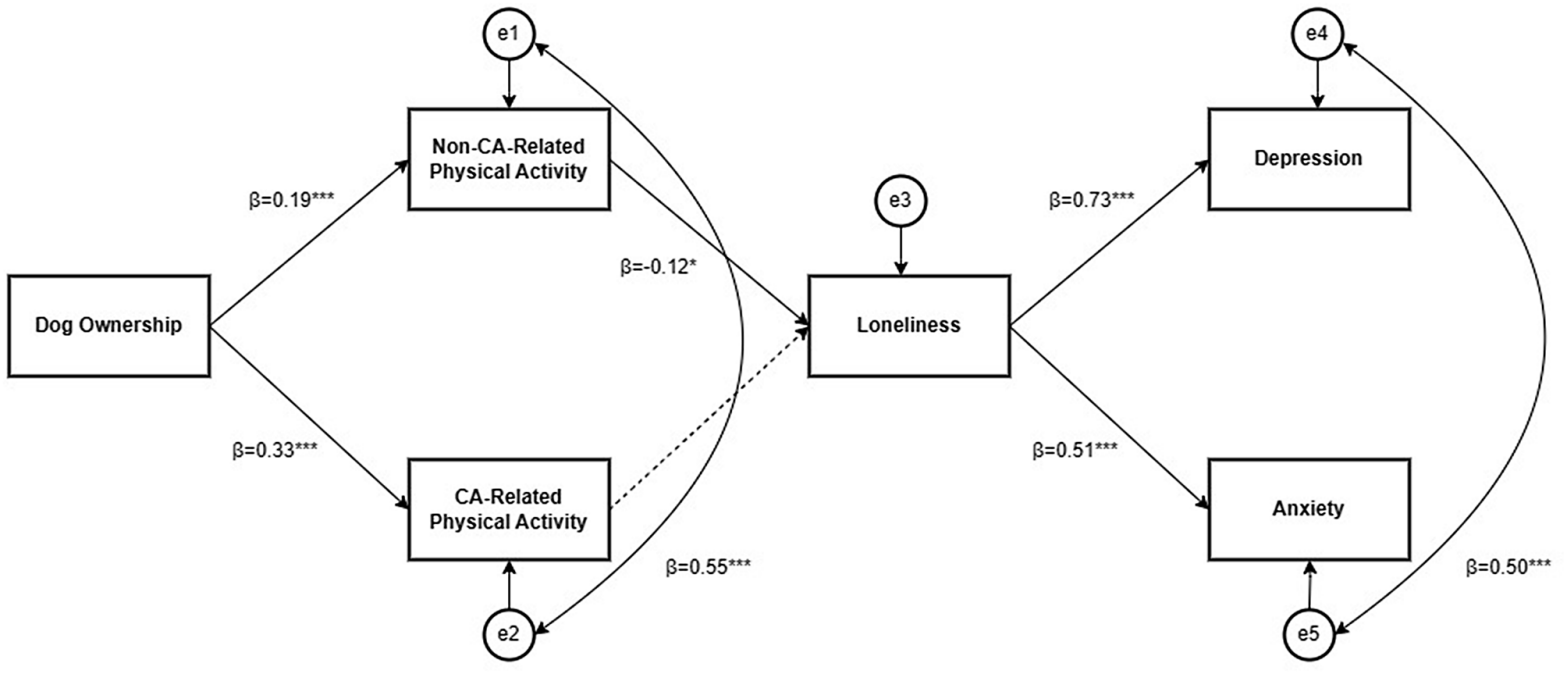
Testing the relationships between dog ownership, non-CA-related physical activity, CA-related physical activity, loneliness, depression, and anxiety, on a sample of CA owners aged under 65 years. *Note*. *** *p* <.001, ** *p*<.01, * *p*<.05. →= Non-significant path. Coefficients are standardised regression weights. CA = Companion animal. RMSEA=0.00; CFI=1.00; IFI=1.00; χ²=6.79, df=8, *p*=.56, χ²/df=0.85. e1 – e5 = residual error terms reflecting the unexplained variance and measurement error for each variable

Figure 1 shows the conceptual model, while Fig. 3 shows the final model with good model fit (RMSEA = 0.00; CFI = 1.00; IFI = 1.00; χ² = 6.79, *df* = 8, *p* = .56, χ²/*df* = 0.85). Table 5 shows the fit statistics for alternative models. The final model (Fig. 3) demonstrates dog ownership was significantly positively associated with non-CA-related physical activity (β = 0.19, *p* < .001), and CA-related physical activity (β = 0.33, *p* < .001), while only non-CA-related physical activity was associated with loneliness (β = -0.12, *p* = .01). Loneliness was significantly positively associated with depression (β = 0.73, *p* < .001), and anxiety (β = 0.51, *p* < .001), with loneliness explaining 53% of the variance in depression (R² = 0.53), and 26% of the variance in anxiety (R² = 0.26). Dog ownership explained 4% of the variance in non-CA-related physical activity (R² = 0.04), and 11% of the variance in CA-related physical activity (R² = 0.11). The error terms for non-CA-related physical activity and CA-related physical activity were significantly positively associated (β = 0.55, *p* < .001) indicating some possible overlap in these constructs.

**Table 5:** Fit statistics for alternative models

Model	χ²	df	*p*	χ²/df	RMSEA	CFI	IFI
1	263.73	9	0.00	29.30	0.27	0.66	0.66
2	263.90	10	0.00	26.39	0.25	0.66	0.66
3	119.88	9	0.00	13.32	0.18	0.85	0.85
4	6.79	8	0.56	0.85	0.00	1.00	1.00

*Note* RMSEA = root mean square error of estimation; CFI = comparative fit index; IFI = incremental fit index

#### Multigroup moderation analyses

The results of the multigroup moderation analyses, comparing each regression path for older adults versus younger adults, are presented in Table 6. The path analyses displayed two significantly different paths, the loneliness to depression path, and the loneliness to anxiety path. The positive relationship of loneliness with depression and anxiety, was stronger for younger adults than for older adults. There was no significant difference between older adults and younger adults on the relationship between non-CA-related physical activity and loneliness, despite path analyses indicating a significant relationship for younger adults, and a non-significant relationship for older adults. This indicates a possible type II error for this path in the older adult path analysis due to a smaller sample size (*n* = 186).

**Table 6:** Multigroup analysis (Chi-Square difference) for loneliness, depression, and anxiety models

Path	65+		U65			
	Estimate	*p*	Estimate	*p*	*z*-score	Result
Dog Ownership →Non-CA-Related Physical Activity	0.49	0.00	0.43	0.00	-0.28	Not Different
Dog Ownership →CA-Related Physical Activity	0.91	0.00	0.77	0.00	-0.74	Not Different
Non-CA-Related Physical Activity →Loneliness	-0.07	0.17	-0.08	0.01	-0.24	Not Different
Loneliness → Depression	1.13	0.00	1.65	0.00	3.99***	Different
Loneliness → Anxiety	0.39	0.00	0.98	0.00	5.46***	Different

*Note* *** *p* < .01; ** *p* < .05

## Discussion

This study examined the relationships between dog ownership, CA related physical activity, and non-CA related physical activity with depression and anxiety via loneliness, within the context of the COVID-19 pandemic. These variables of interest were examined in both older adult CA owners (those aged 65 years and over) and younger adult CA owners (those aged under 65 years). Our study found both older adult and younger adult CA owners experienced similar moderate levels of loneliness, but the positive relationship between loneliness and mental ill health was stronger for younger adults compared to older adults. When considering dog ownership and physical activity, dog owners engaged in higher levels of both CA-related physical activity and non-CA related physical activity, but only non-CA-related physical activity was significantly associated with better mental health outcomes. Bivariate correlations indicated in older adults, non-CA-related physical activity was negatively associated with depression and anxiety, but not associated with loneliness, while in younger adults, non-CA-related physical activity was negatively associated with loneliness and depression, but not associated with anxiety. Overall, in older adults, dog ownership was significantly associated with lower anxiety, but not associated with lower loneliness or depression. In younger adults, dog ownership (compared to ownership of other CA types) was significantly associated with lower loneliness and depression, but not with anxiety.

Our study found older and younger adult dog owners engaged in higher levels of CA-related physical activity than non-dog CA owners. This supports research evidence suggesting the care requirements of dogs leads to increased dog-related physical activity such as dog-walking and playing fetch, activities generally not undertaken with other common CA types [60]. Interestingly, dog ownership was associated with higher levels of physical activity independent of their dog, such as running, strength training, and sports. This indicates people who already have a more active lifestyle may choose to acquire a dog as a CA, as this may be perceived as more able to fit in with their already active lifestyle than other common CA types. This inference is supported by research evidence investigating personality differences between dog and cat owners, with dog owners scoring higher on a measure of ‘liveliness’ [24].

Our findings indicated non-dog related physical activity (such as walking, gardening, running, strength training, and sports) may be more beneficial for mental health than dog-related physical activity, as CA-related physical activity was not associated with mental health outcomes for older adult or younger adult CA owners. When considering the potential differences between these forms of exercise, it is possible that the intensity of playing fetch or walking a dog (walking at the dog’s pace and stopping frequently for the dog to smell objects or play) is lower than that of walking without a dog, gardening, running, strength training, or sports. Dog walking is largely considered light to moderate intensity exercise [49, 58], with research indicating higher intensity exercise provides superior results for mental health compared to lower intensity exercise [56].

In older adult CA owners, non-CA-related physical activity was associated with lower depression and anxiety, but not loneliness, while in younger adult CA owners non-CA-related physical activity was associated with lower loneliness and anxiety, but not depression. This may reflect differences in the nature of the exercise younger adults and older adults engaged in during the pandemic. Some research evidence suggests during COVID-19 older adults preferred to exercise closer to home with the purpose of engaging in physical activity only, rather than incorporating physical activity and social contact due to fears of contracting the virus [46]. While exercise of this nature may have resulted in reduced depression and anxiety via physiological pathways [1] the lack of social contact meant this activity was unlikely to provide a reduction in loneliness.

While we found some evidence for the ‘pet effect’ within dog owners compared to non-dog CA owners, this evidence was mixed and differed based on the age of the subsample, with varied symptoms reduced. These mixed results are consistent with existing mixed CA research conducted during COVID-19 [31]. While we theorised differences in mental health outcomes for dog owners may be due to CA-related physical activity such as dog walking, chance social encounters while walking a dog, and exposure to green spaces while walking a dog, our results indicated it was not this CA-related physical activity associated with mental health outcomes. As our results did not support this hypothesis, other explanations as to why dog ownership may confer better mental health outcomes should be considered. One possible explanation for further investigation is that those with more active lifestyles tend to acquire dogs over other animals as CAs.

### Implications

This study raised interesting findings regarding the relative benefits of dog CA ownership and associated physical exercise compared to other types of CA ownership for loneliness and mental health outcomes, suggesting dog ownership may be more beneficial to reduce symptoms of loneliness, anxiety, depression- with caveats. These caveats relate to the age range of owners, and acknowledgement that there are likely individual differences such as personality traits of dog owners generally that predispose them to both seek out dog CAs over other types, and to engage in exercise regardless of dog ownership. Given the mixed results of this and other studies in HAI, care should be taken by health and other professionals to avoid overstating the benefits of CAO for loneliness and mental health, particularly via increased physical activity associated with animal care, and especially for dogs who have higher care requirements than many other CA types. A person acquiring a dog with the sole purpose of improving their mental health is unlikely to experience mental health benefits. Furthermore, overstating the benefits of dog ownership on this basis poses significant potential welfare risks to individual dogs which may suffer neglect and relinquishment, as well as dog populations which may be bred to meet demand in times of social need, only to be relinquished to shelters or euthanised by veterinarians when no longer desired by ambivalent owners [16]. While the media hype that suggested CAs were beneficial for mental health prompted a boom of CA adoptions during the pandemic [41, 52], in Australia, shelter operators also reported a 30% increase in CA relinquishments after COVID-19 restrictions were lifted in 2021 [16]. This trend is concerning for animal welfare organisations and highlights the ethical dangers in overstating the effects of CAO for loneliness and mental health in humans.

### Strengths, limitations, and future research

This study was the first to investigate both CA-related and non-CA related physical activity and their relationships with mental health among CA owners with dogs, and the first to compare these relationships between older adult and younger adult CA owners with dogs. A large sample of CA owners representing all Australian states and territories was obtained. The varied recruitment methods of this study, via both social media and paper flyer advertisements, supported participation of older adults with varied digital literacy. Participants were able to complete the online and phone survey in the privacy of their own homes, reducing the risk of socially desirable responding.

This study also had some limitations. The sample collected did not include older adults aged 85 years and over, partly due to strict COVID-19 lockdowns for aged care residents preventing recruitment of this group [17]. Novel, unvalidated items developed by the research team were used to measure CA-related physical activity and non-CA related physical activity. Although examples were used to guide responses, some participants may have misinterpreted these items, with the potential for some possible overlap in CA-related and non-CA related physical activity. Further, our sample was skewed towards female participants (76.3%), consistent with the systemic gender skew in HAI research [50]. Caution should be taken when generalising the results of this study to male CA owners. Finally, as this study used a cross-sectional data set, the path analysis was not able to indicate causal effects of the variables of interest on loneliness, depression, and anxiety beyond the theorised temporal order modelled.

Future research in this area should focus on examining additional CA-related variables that may influence the mental health of CA owners, such as personality factors, CA attachment, and burden of CA care. These studies should aim to recruit older adults aged 85 years and over due to high rates of depression and suicidality in this under-researched group [55].

## Conclusion

Dog owners engaged in higher levels of both CA-related physical activity and non-CA-related physical activity, however only non-CA-related physical activity was significantly associated with better mental health outcomes. Older adult CA owners and younger adult CA owners experienced varying effects of dog ownership and non-CA-related physical activity on loneliness, depression, and anxiety. Our findings indicate people who choose to own dogs engage in more active lifestyles, but it is the physical activity they perform independently of their dog that is associated with lower loneliness and better mental health. Members of the public should not be universally encouraged to own a dog in order to support their mental health via increased physical activity.

**Table 7:** Bivariate linear regressions between dog ownership, CA-related physical activity, Non-CA-related physical activity, loneliness, depression, and anxiety

Outcome Measure	Predictor	Older Adults (*n* = 181)					Outcome Measure	Predictor	Younger Adults (*n* = 402)				
		b	SE	*R*²	F(1,180)	*p*			b	SE	*R*²	F(1,401)	*p*
Loneliness	*Constant*	2.09	0.09				Loneliness	*Constant*	2.54	0.06			
	Dog Ownership	0.02	0.11	0.00	0.03	0.87		Dog Ownership	-0.18	0.07	0.02	6.39	0.01
Depression	*Constant*	1.83	0.16				Depression	*Constant*	2.57	0.14			
	Dog Ownership	− 0.09	0.19	0.00	0.22	0.64		Dog Ownership	-0.39	0.16	0.01	5.65	0.02
Anxiety	*Constant*	1.42	0.09				Anxiety	*Constant*	2.00	0.12			
	Dog Ownership	-0.24	0.11	0.02	4.77	0.03		Dog Ownership	-0.23	0.14	0.01	2.86	0.09
CA-Related Physical Activity	*Constant*	2.29	0.14				CA-Related Physical Activity	*Constant*	2.17	0.09			
	Dog Ownership	0.91	0.17	0.14	29.20	0.00		Dog Ownership	0.77	0.11	0.11	47.30	< 0.001
Non-CA-Related Physical Activity	*Constant*	2.65	0.14				Non-CA-Related Physical Activity	*Constant*	2.46	0.09			
	Dog Ownership	0.49	0.16	0.05	8.73	0.00		Dog Ownership	0.43	0.11	0.04	14.52	< 0.001
Loneliness	*Constant*	2.06	0.14				Loneliness	*Constant*	2.56	0.09			
	CA-Related Physical Activity	0.01	0.04	0.00	0.07	0.79		CA-Related Physical Activity	-0.05	0.03	0.01	3.12	0.08
Depression	*Constant*	2.00	0.25				Depression	*Constant*	2.51	0.20			
	CA-Related Physical Activity	-0.08	0.08	0.01	1.04	0.31		CA-Related Physical Activity	-0.08	0.07	0.00	1.20	0.28
Anxiety	*Constant*	1.36	0.14				Anxiety	*Constant*	1.87	0.17			
	CA-Related Physical Activity	-0.04	0.05	0.00	0.68	0.41		CA-Related Physical Activity	-0.03	0.06	0.00	0.27	0.61
Loneliness	*Constant*	2.29	0.15				Loneliness	*Constant*	2.63	0.09			
	Non-CA-Related Physical Activity	-0.07	0.05	0.01	1.90	0.17		Non-CA-Related Physical Activity	-0.08	0.03	0.02	6.29	0.01
Depression	*Constant*	2.40	0.27				Depression	*Constant*	2.75	0.21			
	Non-CA-Related Physical Activity	-0.21	0.08	0.04	6.45	0.01		Non-CA-Related Physical Activity	-0.17	0.07	0.01	5.42	0.02
Anxiety	*Constant*	1.64	0.15				Anxiety	*Constant*	2.00	0.18			
	Non-CA-Related Physical Activity	-0.13	0.05	0.04	7.10	0.01		Non-CA-Related Physical Activity	-0.08	0.06	0.00	1.65	0.20

*Note b* = unstandardised coefficient; *SE* = standard error; CA = companion animal

## Electronic supplementary material

Below is the link to the electronic supplementary material.

Supplementary Material 1

## Data Availability

Data used for this study is available upon request of the corresponding author and following approval by the Deakin University Human Research Ethics Committee.

## Appendix A

### Supplementary Information

#### Post-hoc analyses

Given the significant unique associations between dog ownership, CA-related physical activity, and non-CA-related physical activity on outcome measures shown in bivariate correlations, but the broken pathways between dog ownership, CA-related physical activity, and non-CA-related physical activity and outcome measures in path analyses, a series of bivariate linear regressions were conducted to clarify the relationships between dog ownership, non-CA-related physical activity, CA-related physical activity, loneliness, depression, and anxiety in older adult and younger adult CA owners. For a linear bivariate regression (two-tailed) with one group for *a*=0.05 and 1-β =0.80, an a priori statistical power analysis indicated *N*=171 would detect a slope of 0.21. Table 7 displays the results of the bivariate linear regressions. As shown, in older adults, dog ownership (versus ownership of another CA type) is significantly associated with lower anxiety, explaining 2% of the variation in anxiety (R²=0.02) but not loneliness or depression. Conversely, in younger adults dog ownership is significantly associated with lower loneliness and depression, explaining 1 to 2% of the variation in mental health outcomes, but not significantly associated with anxiety. In older adults, non-CA-related physical activity was significantly associated with lower depression and anxiety, each accounting for 4% of the variation in mental health outcomes, but not significantly associated with loneliness. In younger adults, non-CA-related physical activity was significantly associated with lower loneliness and depression accounting for 1 to 2% of the variation in mental health outcomes, but not significantly associated with anxiety. CA-related-physical activity was not significantly associated with mental health outcomes in either older adults or younger adult CA owners.
