# Supplementary material for: Dog ownership, physical activity, loneliness and mental health: a comparison of older adult and younger adult companion animal owners
Source: BMC Psychol. 2024 Nov 1;12:618. doi: 10.1186/s40359-024-02104-x (PMC11529494; doi:10.1186/s40359-024-02104-x)
Supplement: Supplementary file 1 — Supplementary Material 1 [file 40359_2024_2104_MOESM1_ESM.docx]

**Supplementary Information**

**Novel Measure of CA-Related Physical Activity and Non-CA Related Physical Activity**

Physical activity was measured in the current study titled ‘Dog Ownership, Physical Activity, Loneliness and Mental Health: A Comparison of Older Adult and Younger Adult Companion Animal Owners’ using two original items developed by the research team (Zablan et al., 2020). CA-related physical activity was measured using the following item: ‘*Approximately how many hours of pet-related physical activity do you participate in per week? (e.g., walking a dog, cleaning up after your pet etc.)’.* Non-CA-related physical activity was measured using the following item: *‘Approximately how many hours of physical activity do you participate in per week? (e.g., walking, gardening, running, strength exercises, sports etc.)’.* Participants responded to each item on a 4-point Likert scale (1 = ‘less than one hour’, 2 = ‘1 to 3 hours’, 3 = ‘3 to 6 hours’, 4 = ‘more than 6 hours’).
